# Supplementary material for: Are carbon emissions trading and green financial instruments synergistic? -Comprehensive quantitative research based on content analysis
Source: PLoS One. 2024 Mar 7;19(3):e0298601. doi: 10.1371/journal.pone.0298601 (PMC10919601; doi:10.1371/journal.pone.0298601)
Supplement: S1 Data — (ZIP) [file pone.0298601.s001.zip › Data/地级市Z_F工作报告.zip!/║╙─╧/Σ≡║╙╩╨/└┤╫╘╣1⁄2╓┌║┼í╛┬φ┐╦╩2╛▌═°í┐_2015.docx]

各位代表：

现在，我代表漯河市市人民政府，向大会报告工作，请予审议，并请各位政协委员和其他列席人士提出意见。

一、2014年工作回顾

过去的一年，漯河市政府在省委、省政府和市委的正确领导下，在漯河市人大、市政协的监督支持下，和漯河市人民一道，团结拼搏，实干创新，漯河市经济社会发展取得新成效。

———漯河市经济平稳增长。初步预计，漯河市生产总值952.3亿元，增长9.2%、居全省第8位；规模以上工业增加值555.3亿元，增长12%、居全省第5位；固定资产投资773.3亿元，增长19.3%、居全省第8位；社会消费品零售总额380.2亿元，增长13.1%、居全省第2位；地方公共财政预算收入62.9亿元，增长16.4%、居全省第7位；城镇居民人均可支配收入23281元，增长10%、居全省第4位；农民人均纯收入10893元，增长10.3%、居全省第6位；金融机构人民币贷款余额420.2亿元，比年初增加76.3亿元、增长22.2%，贷款增量创历史最好水平、增幅居全省第3位。

———产业结构调整步伐加快。主导产业进一步壮大，新兴产业快速发展。规模以上食品工业主营业务收入1549亿元，占漯河市规模以上工业的60%；高新技术产业增加值84亿元，增长36.9%。工业1351工程扎实推进，新增规模以上工业企业63家。双汇集团荣获省长质量奖，新培育国家级高新技术企业3家，新建省级工程技术研究中心52家，新增省级创新型企业3家。商务中心区、特色商业区建设实现新突破，现代物流园区、城市综合体等一批服务业重点项目加快推进，电子商务等新兴业态蓬勃发展，服务业发展水平明显提升。

———发展动力和后劲持续增强。简政放权力度加大，分两批取消、下放423项行政审批事项和具有审批性质的管理事项；工商登记制度改革激发了全民创业活力，漯河市新登记注册市场主体1.4万户，其中，新登记注册企业3294户，增长75.5%。2.5亿元规模的生物医药创投基金获得批复，小微企业发展基金挂牌运营，食品产业基金顺利启动，万洲国际在香港联交所主板成功上市。31家企业实施了战略重组，引进战略投资33.6亿元。开放招商成果丰硕，成功举办了第十二届中国(漯河)食品博览会、第十四届全国“村长”论坛和首都经济圈推介会等多项专题招商活动，招引了一批重大项目；实际利用外资7.9亿美元，总量居全省第4位；实际利用省外资金192.2亿元，增长15.7%；出口总额5.2亿美元，增长104.8%、居全省第1位。重点项目建设力度加大，市定150个重点项目完成投资313亿元。产业集聚效应更加明显，漯河市6个产业集聚区实现主营业务收入1700亿元，从业人员达到20万人。临颍县产业集聚区被评为全省“十先”、升格为省级经济技术开发区，经济技术产业集聚区摘取“二星”，漯河市星级产业集聚区占比居全省前列。

———现代农业发展成效明显。漯河市被农业部确定为国家现代农业示范区。全年投入和发放各类涉农资金25.4亿元。新建成高标准粮田36万亩。粮食总产174.8万吨，实现“十一连增”。新培育省级农业产业化龙头企业7家、农业产业化集群8个，新增农民专业合作社和家庭农场740家。蔬菜种植面积97.4万亩，新创建6个国家级蔬菜示范园区。完成植树造林面积5.2万亩。

新增农牧一体的生态循环养殖场28个，漯河市被确定为全省生态循环畜牧业试验示范市。综合农业机械化水平达到84.5%，高于全省平均水平8.5个百分点，夏秋两季秸秆均实现全面禁烧。农田水利建设力度加大，新发展有效灌溉面积10.2万亩。农村土地流转面积122.1万亩，占家庭联产承包经营总面积的52.9%、居全省第2位。

———城乡建设力度加大。新型城镇化建设实现新突破，初步预计，漯河市城镇化率达到45.7%，比上年提高1.5个百分点。新修编的城市总体规划获得省政府批复，17项城市专项规划编制基本完成。156个城建项目扎实推进，完成投资230亿元，是历年来城市建设投入力度最大、项目建设最多的一年。城中村和旧城区改造稳步推进，完成房屋征收面积200万平方米，开工建设安置房面积280万平方米。市城乡一体化示范区确立了发展定位和总体布局，管理体制得到理顺。西城区开发建设步伐加快，商务中心区重大项目推进顺利。“创卫”工作攻坚破难，已取得阶段性成果，城市形象明显提升。

S330等干线公路完成大修改造，漯河沙河航运港建设快速推进。临颍、舞阳两个县城建设力度加大，小城镇和新农村建设扎实推进。新解决农村27万人饮水安全问题，新建、改造农村公路196公里。农村人居环境综合整治成效明显。成功创建2个省级生态乡镇、13个省级生态村。17个贫困村整村推进任务全面完成，实现2.4万农村贫困人口稳定脱贫。 ———民生质量得到提高。城镇新增就业5.7万人、再就业2万人，新增农村劳动力转移就业3.7万人。社会保险基本实现全覆盖，农村居民大病医疗保险工作顺利启动，企业退休人员养老金、城乡低保和农村五保户供养标准、城镇职工基本医疗保险最高支付限额、新农合补助标准均得到提高。使用8.5亿元住房公积金，帮助5000户住房困难职工改善了居住条件。新开工建设保障性住房2.7万套，在全省率先实现公共租赁住房常态化分配。

社会事业全面发展。漯河市临颍县、舞阳县、郾城区通过国家义务教育均衡发展评估验收，筹建、改扩建9所中小学，“大班额”问题得到有效缓解。普通高考成绩喜人，漯河高中等学校升入名牌大学的人数大幅增加。职业教育、高等教育积极稳步发展，漯河医专、漯河职院加强软硬件建设，学校竞争力明显提升；漯河技师学院、漯河食品职业学院、市第一中专快速发展，势头很好。城乡医疗卫生服务体系进一步完善，县级公立医院改革稳步推进。“幸福漯河”系列文化活动入围全省第一批公共文化服务体系示范项目。新增3个国家4A级景区。举办了8项省级以上高水平体育赛事，全民健身示范市创建工作全面启动，为300个行政村安装了体育健身设施。养老服务业健康发展，漯河市被确定为全国养老服务业综合改革试点市。漯河市市区便民自行车慢行系统投入使用。更新公交车辆120台，市内公交车IC卡实现全覆盖侯卫东官场笔记。计划生育、环境保护和土地管理三大基本国策全面落实，更加重视生态环境建设，蓝天工程、碧水工程推进有力，治理水污染、大气污染、粉尘污染成效初显，漯河市城市河流治理满意度测评居全省第1位，城市环境空气质量居全省前列。切实加强土地报批，确保了重点工程和民生项目用地需求。进一步强化了审计监督，对217个单位开展了审计。统计、气象等工作得到加强，广播影视、新闻出版、外事侨务、民族宗教、史志档案、慈善事业等取得新成效。妇女、未成年人、老年人和残疾人合法权益得到保障。国防教育、双拥工作和人民防空建设继续加强，军民融合式发展进一步深化，驻漯解放军、武警消防官兵和民兵预备役人员在支持地方建设中作出了重要贡献。

社会大局和谐稳定。民主法制建设不断加强，自觉接受人大和政协监督，665件人大代表议案、建议和政协提案全部办结，依法治市和法治政府建设深入推进，完成了第八届村委会换届选举工作。廉政建设力度加大，政风行风持续好转。精神文明建设成效显著。深入开展党的群众路线教育实践活动，着力解决了“四风”突出问题。

信访和安全生产形势持续平稳，食品药品监管水平进一步提高，平安漯河建设深入推进，应急管理工作得到加强。漯河市荣获全省首批依法行政示范市、全省平安建设先进市等称号。

各位代表!过去的一年，是极不寻常的一年，宏观经济进入新常态，经济下行压力加大，漯河市不可能独善其身。面对多重困难压力和严峻挑战，我们清醒地认识到，漯河虽然发展较快，但块头不大、实力不强，在这爬坡过坎的关键时期，如果稍有懈怠，就会丧失机遇，就会被新一轮发展的列车甩在后头。作为负责任的政府，必须敢于担当，积极作为，在市委领导下，强力推进六项重大攻坚任务，不管形势多么复杂多变，始终坚持发展第一，任尔东西南北风，咬定发展不放松。一年来，在经济运行、城市建设、招商引资、民生改善等方面，我们攻克了一批批难题，取得了明显成效。过去一年的工作实践，我们深深感到，人民群众的信任和期盼，是做好政府工作的不竭动力；科学谋划、统筹兼顾、迎难而上、坚韧不拔，是有效推进工作的有力保障。

一年来，在经济下行压力加大中，我们坚定不移抓项目、备后劲。坚持以项目建设为中心，充分发挥投资对经济增长的关键性作用，建立了项目建设专项述职制度，坚持实行重大项目领导分包、重点项目例会、联审联批等制度，及时协调解决项目推进中的重大问题，多次组织重点项目集中开工、观摩讲评活动，强力推进项目建设，持续掀起项目建设高潮，一大批项目快速推进，57个重点项目建成投运，拉动了经济增长，增强了发展后劲。

一年来，在加快产业转型升级中，我们坚定不移提质量、增效益。坚持速度与质量并重，更加注重发展质量，在关停、拆除10家高污染、高耗能企业的同时，培育壮大了一批发展快、效益好、潜力大的中小企业，优化了产业结构，提高了经济增长质量，漯河市工业用电量增长11.1%、居全省第3位，税收收入占地方公共财政预算收入的比重达到78.3%、居全省第1位。

一年来，在日益激烈的区域竞争中，我们坚定不移强县区、壮实力。坚持把县区经济作为支撑漯河市经济发展的顶梁柱，大力支持，强化指导，促进各县区高树标杆、加压驱动、明争暗赛、竞相发展，县区经济实力明显增强，有4个县区的公共财政预算收入增速达到20%以上，有3个县区的公共财政预算收入增速在全省158个县区排序中进入前40名。

一年来，在财政收支矛盾加剧中，我们坚定不移惠民生、促和谐。坚持发展与民生并重，更加注重改善民生，持续加大民生投入，漯河市各级财政用于民生的支出首次突破100亿元、达到101.4亿元，占地方公共财政预算支出的74.8%、比上年提高了2.3个百分点，省定十项民生工程、市定十件民生实事较好完成，市区背街小巷改造全面完成、老旧小区全部整治，农村人居环境明显改善，就医、就学、就业、困难群体生活保障等一批热点难点问题得到有效解决，人民群众享受到更多实惠。

各位代表!过去的一年，我们能取得这些成绩确实不易。这是省委、省政府和市委正确领导的结果，是漯河市人大、市政协监督支持的结果，是漯河市人民团结奋斗的结果。在此，我代表市人民政府，向工作在各条战线上的工人、农民、知识分子、干部，向驻漯解放军、武警消防官兵、公安民警以及各界人士，致以崇高的敬意!向所有关心支持漯河发展的同志们、朋友们，表示诚挚的感谢!各位代表!在看到成绩的同时，我们也清醒地认识到，漯河市经济社会发展中还存在不少问题，政府工作与人民群众的期望还有一定差距。受宏观经济形势的影响，漯河市造纸、纺织等传统行业产能过剩，部分企业产品结构调整较慢，生产经营极为困难，给本来就总量不大的漯河市工业经济带来很大的负面冲击，成为漯河市经济发展的拖累点，虽然高新技术企业成为新的增长点，但毕竟支撑作用有限，增长点尚消化不了拖累点，致使漯河市经济增长与年初预期目标还有一些差距；财政收入增速放缓，多种刚性支出增加，收支矛盾突出；产业层次总体不高、整体竞争力不强，食品产业规模大但附加值低，电子信息、生物医药等新兴产业发展快但规模小，服务业发展水平需要进一步提升；骨干企业群体不大，梯次结构不合理；教育、文化、医疗卫生等民生领域投入还需要持续加大，安全生产、涉法信访、征地拆迁、生态环保等方面还存在一些薄弱环节；政府职能转变、简政放权还不到位，有的地方和一些部门的工作人员思想观念、工作作风、能力素质与经济发展新常态的要求还不相适应。对此，我们一定高度重视，认真对待，针对存在的问题，逐一研究改进和解决措施，决心在新的一年里开创政府工作新局面!二、2015年工作安排

今年是全面深化改革的关键之年、全面推进依法治国的开局之年、全面完成“十二五”规划的收官之年，也是漯河市深入推进六项重大攻坚任务的重要之年。当前，经济发展进入新常态，对政府工作提出了新的更高要求，我们必须认清形势、把握大势。一是在适应新常态中找准结合点。新常态下，我国的经济增速正从高速增长转向中高速增长，经济发展方式正从规模速度型粗放增长转向质量效率型集约增长，经济结构正从增量扩能为主转向调整存量、做优增量并存的深度调整，经济发展动力正从传统增长点转向新的增长点。我们必须认识新常态、适应新常态、引领新常态，更加自觉地坚持以提高经济发展质量和效益为中心，大力推进经济结构战略性调整，更好地发挥消费的基础作用和投资的关键作用，更加注重科技进步和改革创新，着力培育新的经济增长点，持续增强经济发展的内生动力。二是在迎接新挑战中找准突破点。面对来自经济增长速度的挑战，我们必须保持经济增速高于全省平均水平，以速度上的领先弥补经济总量上的差距；面对发展动力转换的挑战，我们必须在坚持把投资作为拉动经济增长主动力的同时，在创新能力上求突破，以创新推动发展；面对来自区域竞争的挑战，我们必须发挥比较优势、增创新的优势，确保在激烈的区域竞争中立于不败之地。三是在抢抓新机遇中找准关键点。国家继续实施积极的财政政策和稳健的货币政策，全面深化改革的力度进一步加大，粮食生产核心区、中原经济区、郑州航空港经济综合实验区三大国家战略的深入实施，我省建设先进制造业大省、高成长服务业大省、现代农业大省的扎实推进，有利于我们争取上级支持、吸引外来投资，有利于不断激发市场发展活力，有利于漯河市持续做大做强食品、化工等主导产业，培育壮大电子信息、生物医药等战略性新兴产业，加快发展服务业和现代农业。我们面临的机遇和挑战并存，既要正视困难、坚定信心，更要抢抓机遇、乘势而上，推动漯河市经济社会发展再上新台阶。

今年政府工作的总体要求是：全面贯彻落实党的十八届三中、四中全会和省委、市委经济工作会议精神，以稳中求进、进中提升为总基调，以深化六项重大攻坚任务为统揽，以项目建设为中心，着力稳增长、调结构、强支撑、促改革、惠民生，促进经济持续平稳较快发展、社会大局和谐稳定。

经济社会发展的主要预期目标是：生产总值增长8.5%，在实际工作中努力争取更好结果；规模以上工业增加值增长11%以上；一般公共预算收入增长9%；固定资产投资增长17%；社会消费品零售总额增长13%；城镇居民人均可支配收入、农民人均纯收入均增长9%左右；节能减排和环境质量改善完成省下达任务。

实现上述目标，重点抓好六项工作。

(一)更加注重稳增长、调结构，着力加快产业转型升级。坚持做大做强主导产业、大力培育新兴产业、不断优化产业结构，提高经济整体竞争力。

-深入实施工业1351工程。对纳入1351工程的企业，重点服务，大力支持，帮助企业破瓶颈、拓市场、上项目、壮实力，加快培育结构合理的骨干企业梯队。推进工业化和信息化深度融合，强化企业创新主体作用。

力争新培育5家国家级高新技术企业，新建3个省级工程技术研究中心，积极组建产业技术创新战略联盟。加快企业上市步伐，重点培育30家上市后备企业，努力实现企业上市新突破。

持续优化工业结构。抓好中国食品百强工业园、临颍休闲食品基地建设，发挥双汇等龙头企业的带动作用，推动食品产业高端化发展，增强漯河市食品产业的领军作用。支持盐化工、钛化工企业开发高附加值产品，大力发展精细化工。以东兴电子产业园、漯河健康产业园、沙澧医疗器械产业园为载体，加快建设全省电子信息和生物医药产业基地。

推动造纸、机械、制鞋、纺织等传统产业技术改造和产品研发，加快转型升级，使其焕发新的生机和活力。

提升产业集聚区发展水平。按照每个产业集聚区不同功能定位，分类入驻项目，提高投资强度，加快产业集聚。今年，漯河市6个产业集聚区完成固定资产投资500亿元，规模以上企业主营业务收入达到2000亿元。强化综合功能，抓好投融资、劳动保障、创新研发、土地整理储备等平台建设，完善产业集聚区基础设施和配套服务设施，加快产业集聚区内的村庄撤村建居步伐，实现产城融合发展，促进产业集聚区向省级经济技术开发区提升。着力培育临颍现代家居产业园、郾城漯西精细化工产业园、召陵钛化工产业园等专业园区，努力促使其尽快发展成为产业集聚区。

加快发展服务业。强力推进商务中心区和特色商业区建设，重点抓好闽商大厦、天翼滨湖国际广场等14个项目建设，推动商务中心区早出形象；着力推进城市综合体、商业广场等一批重点项目，尽快打造特色商业区的特色和亮点。大力发展现代物流业，提升发展商贸业，培育发展电子商务等新兴业态，做大做强阿里巴巴漯河产业带，招引电子商务、速递物流企业建设区域分拨配送中心，抓好双汇全国冷链物流网升级、沙河港物流园区、宏进农副产品国际物流中心、闽豫钢材物流港、瑞通国际汽配建材城、义乌国际商贸城等一批重点项目，努力打造冷链、粮食、汽贸、家电等物流品牌，加快建设区域性现代商贸物流中心。积极发展金融保险、健康养老、房地产等服务业，推动服务业提速度、扩规模、上水平。

(二)更加注重招大商、上项目，着力抓好投资拉动。坚持锲而不舍强招商、全力以赴抓项目，为漯河市经济社会发展备足后劲。强力开放招商。突出招大引强，重点围绕食品、化工、电子信息、生物医药等产业，瞄准世界500强、国内500强及行业百强，引进一批发展前景好、投资强度大、科技含量高、财税贡献多的大项目。强化集群招商，开展延链、补链招商，引进上下游关联配套企业 诛仙小说。充分发挥企业的招商主体作用，大力开展以商招商。精心组织好第十三届中国(漯河)食品博览会，深入开展各种专题招商活动，新签约总投资超亿元项目达到100个以上。拓宽开放领域，加快推进现代服务业、现代农业、社会事业、城镇基础设施、节能减排等领域的开放招商。完善开放平台，建成运营进口肉类指定口岸漯河查验区。加快培育新的外贸进出口增长点。

狠抓项目建设。全年安排重点项目154个，计划完成投资372亿元，带动全社会固定资产投资完成900亿元以上。强化项目建设和管理，推动项目早开工、早投产、早见效。重点抓好双汇第二工业园二期、旺旺新工业园、南街村食品工业园、三剑客乳品工业园、淞江医药产业园、河南大地集团白酒酿造等50个项目竣工投产。加强与省直部门的联系和对接，争取上级更多的资金和项目，争取省里的新型城镇化建设基金和产业发展基金投向漯河，争取省里的产业发展试点布局漯河。结合“十三五”规划编制，抓紧谋划储备一批重大项目，增强投资后续支撑能力。

(三)更加注重强基础、创特色，着力发展现代农业。坚持以建设国家现代农业示范区为统领，加快转变农业发展方式，不断提升农业现代化水平。

持续推进高标准粮田建设。搞好技术、服务跟进，促进良种、良法配套，确保软件、硬件同步推进。重点抓好舞阳泥河洼16万亩和临颍、郾城两个10万亩高标准粮田建设。

漯河市新建高标准粮田10万亩，稳定提高粮食综合生产能力。

积极培育现代农业产业化集群。以培育壮大农业产业化龙头企业为重点，加快发展“龙头企业+基地+合作社+农户”模式的农业产业化集群，引领漯河市现代农业发展。

深入实施双汇产业化提升行动，依托规模养殖场，着力发展农牧结合的生态循环畜牧业，全年新培育生态规模养殖场20个以上。

严格农产品质量安全管理，加快培育一批农业品牌。

大力发展都市生态农业。坚持生产基地化、基地园区化、园区景点化，以城区周边乡镇为重点，因地制宜发展生态农业、设施农业、休闲农业、观光农业，建设以蔬菜、林果、苗木花卉等为主的现代都市生态农业示范园区。

加快构建新型农业经营体系。在信贷、保险和涉农项目安排等方面，加大对新型农业经营主体的支持力度。全年新发展种养大户1000家以上、农民专业合作社200家以上、家庭农场100家以上。稳妥推进农村土地有序流转。加大新型职业农民培训力度。建立多层次、多形式、多元化的农业社会化服务体系。

(四)更加注重抓统筹、促协调，着力推动新型城镇化建设。坚持城乡统筹发展，持续推进中心城市、县城、中心镇和新农村建设，不断提高城镇化质量。

突出规划引领。抓好城市专项规划和特色风貌规划编制，优化城市基础设施和公共设施布局，加快中心城区建筑高度、建设强度规划，扎实推进村镇体系规划编制。强化规划执法，深入开展违法占地、违法建设整治，坚决维护规划的严肃性和权威性。

强化中心城市建设。推进“五角场”、东外滩、荣昌广场、城市场馆、会展中心五大节点，人民路城市综合体、奥特莱斯商业街、龙江生态城等八大街区建设，努力打造城市特色节点和时尚街区。加快推进中山路上跨立交桥、解放路路桥建设工程、沙澧连通桥闸工程、太白山路贯通工程、提升改造龙江路等十大城建工程，完善城市功能，丰富城市内涵。着力实施公租房建设、市场建设改造、城区居民安全饮水等十项惠民工程，切实解决群众关注的热点问题。积极推进旧城区和城中村改造，着力抓好房屋征收和安置房建设，加快改善人居环境。坚持优先发展公交，加快公交场站建设，提升公交服务水平。加大“黑出租”、“摩的”治理力度，规范市区交通秩序，努力解决市区停车难、交通拥堵等问题。深入推进“创卫”工作，加强城市精细化管理，力争如期成功创建国家卫生城市。全面开展全国文明城市创建活动。围绕市城乡一体化示范区发展定位和总体布局，完善提升规划体系，引进战略投资者，尽快启动起步区开发建设，加快建设新型工业化示范区、都市生态农业示范园区、临港产业园、区域高端服务区和文化教育科技产业园区。着力打造经济技术开发区软硬环境，提升国家级开发区形象。加大西城区基础设施和配套服务设施建设力度，尽快培育产业，努力建成真正意义上的漯河新城区。

加快县城和小城镇建设。推进临颍、舞阳两个县城与中心城市组团发展，优化中心城市与两县的产业布局，形成分工合理、集群发展格局。完善县城基础设施和公共服务设施，加快推进S238、S330改造等交通项目，形成快速交通体系，促进县城与中心城市融合发展。筛选一批基础好、潜力大的特色乡镇，制定发展规划，加大支持力度，强化产业培育，完善镇区基础设施，加快撤乡建镇步伐，打造一批宜工、宜农、宜商、宜游的特色小城镇。

分类推进新农村建设。持续抓好新型农村社区建设，加强对现有建设试点的分类指导，着力抓好基础配套建设，促进社区可持续发展。完成16个贫困村整村推进建设任务，再实现1.8万农村贫困人口稳定脱贫。

南水北调配套工程建成投用。抓好农田水利建设和中小河流域治理，新打机井2700眼、维修机井6000眼。加快农村基础设施建设，新建、改造农村公路300公里以上，新解决17万人的饮水安全问题，新增沼气用户2300户。加快美丽宜居乡村建设，深入开展农村人居环境综合整治，探索农村环境卫生长效保洁管护机制，争创一批省级农村人居环境达标村、示范村，进一步改善农村生产生活条件。(五)更加注重激活力、添动力，着力深化改革创新。坚持把改革创新贯穿经济社会发展各个领域，统筹推进，重点突破，努力取得更大成效。

深化行政体制改革。进一步简政放权，把政府不该管的彻底放开、放活，把该管的坚决管住、管好。深入推进行政审批制度改革，继续取消、下放行政审批事项，扎实开展行政审批标准化试点。全面清理行政权力，公开政府部门权力清单。完成市、县政府机构改革，推进事业单位分类改革。完成党政机关公务用车制度改革。

深化财税体制和金融改革。完善政府预算体系，全面推进预决算公开。强化税收征管，清理规范税收等优惠政策。加强地方性债务管理，积极推行政府与社会资本合作模式，支持政府投融资公司多元化融资，加快农业投融资平台建设 完美世界小说。大力支持各商业银行和政策性银行发展，积极引进股份制商业银行，加快设立财务公司等新型金融机构。

推进农信社改制组建农商行工作。加强金融风险监测预警，严厉打击非法集资等扰乱金融秩序行为，打造更加宽松的金融生态环境。

深化其它领域改革。积极发展混合所有制经济，鼓励非公有制企业进入特许经营领域，支持骨干企业战略重组。完善农村土地管理制度，加快宅基地使用权和集体建设用地使用权等确权登记办证工作。抓好交通运输管理体制和交通综合执法体制改革，继续推进户籍制度、社会事业、生态环保、住房公积金等各领域改革，释放更多改革红利。

实施创新驱动。切实加强观念创新、思维方式创新和体制机制创新，加快科技创新，积极开展产品创新、业态创新、商业模式创新，大力培育新的增长动力。

(六)更加注重惠民生、增福祉，着力提升群众幸福指数。坚持把保障和改善民生作为政府一切工作的出发点和落脚点，持续加大民生投入，认真办好省定十项民生工程和市定15件民生实事，尽最大努力让发展成果更多更公平地惠及漯河市人民。

抓好就业创业工作。加快创业型城市创建工作，健全就业创业服务体系，以创业促进就业，深入推进全民技能振兴工程，推动实现高质量就业。重点做好高校毕业生、农村转移劳动力、城镇困难人员、退役军人就业工作。新增城镇就业4万人，新增农村劳动力转移就业2.5万人，动态消除“零就业家庭”。

完善社会保障体系。提高企业退休人员基本养老金、机关事业单位工作人员基本工资标准、农村五保户供养标准、城乡居民基础养老金标准、新农合和城镇居民医保补助标准、基本公共卫生服务经费补助标准，全面实施城镇居民大病保险，健全重特大疾病保障制度，落实市疾病应急救助基金管理实施办法，实现城乡居民与城镇职工之间养老保险的转移接续，全面实施临时救助制度，建立健全老年福利制度，加快建设社会福利中心，扎实推进社会保障一卡通。加大棚户区改造力度，加快保障性住房建设，开工建设各类保障性住房8016套。进一步完善社会养老服务体系、社会救助体系和社会福利体系，切实做好全国养老服务业综合改革试点工作，开工建设市养老产业园，积极探索多元化社会养老新路子。

加快发展社会事业。努力办好人民满意的教育。实施第二期学前教育三年行动计划；建成4所中小学，筹建、改扩建29所中小学，扩充城镇义务教育资源，推动义务教育均衡发展；在新建大型社区、城市综合体等区域，按照规划要求，配套建设幼儿园和中小学；提升高中教育质量；实施职业教育攻坚二期工程，大力支持漯河医专升本，积极推进漯河职院转型提质、与本地经济融合发展，加快漯河技师学院高技能人才培养基地建设，鼓励漯河食品职业学院等学校加快发展。完善医疗卫生服务体系，抓好市中心医院西城区分院、全科医生临床培养基地、市第二人民医院和市第六人民医院病房大楼、县区中医院业务用房和疾病预防控制中心等项目建设，做好重大传染病防控救治工作，全面推进县级公立医院综合改革，加快推进社会办医。坚持计划生育基本国策不动摇，促进人口长期均衡发展。加快发展文化旅游业，以许慎文化、贾湖文化、舞阳农民画、沙澧文化超市、幸福漯河健康舞等为重点，打响特色文化品牌；加大文化惠民力度，新建680个农村综合性文化服务中心，健全完善公共文化服务体系，加快城市展示馆、市图书馆、市博物馆、市群艺馆建设，开工建设市民之家、市青少年综合实践基地等重大项目，积极推进中国文化研究院漯河国际交流中心等项目；启动开源景区创建国家5A级旅游景区工作。大力培育社会主义核心价值观，深入推进公民道德建设，广泛开展群众性精神文明活动，提升全民文明素质。举办高水平体育赛事，深入开展全民健身示范市创建工作，实施100个行政村农民体育健身工程。支持妇女、儿童、残疾人、红十字、慈善、老龄等事业发展，新建100所留守儿童之家。做好统计、气象、广播电视、新闻出版、外事侨务、民族宗教、史志档案等工作。支持工会、共青团、妇联等人民团体发挥桥梁纽带作用。加强国防教育和国防后备力量建设，支持驻漯解放军、武警部队、预备役部队、民兵和人民防空建设。

深入开展双拥共建，积极创建新一届全国双拥模范城，进一步巩固和发展军政军民团结。

加强生态环境保护。坚持源头严防、过程严管、后果严惩，以水污染和大气污染防治为重点，深入实施蓝天工程、碧水工程、乡村清洁工程和城市河流清洁行动计划，进一步加快城市建成区工业企业“退二进三”、“退城入园”步伐，切实抓好农村面源污染治理，持续推进林业生态市建设提升工程，着力打造天更蓝、水更清、地更绿的良好生态环境。

强化社会治理创新。加强城市社区建设，创新社区管理服务。认真做好信访稳定工作，推行逐级信访制度，完善社会稳定风险评估机制。强化安全生产和食品药品安全监管，防止重特大安全事故发生。完善立体化社会治安防治体系，严厉打击各类违法犯罪活动，健全反恐工作机制，深化平安漯河建设，切实加强应急管理，确保漯河市社会大局持续和谐稳定。

在做好以上重点工作的同时，坚持用改革的精神、创新的理念和科学的方法，研究重大问题，谋划重大项目，精心编制“十三五”规划，为漯河未来发展描绘好宏伟蓝图。

三、全面加强政府自身建设

面对新形势、新常态、新任务，必须更加注重政府自身建设，巩固扩大党的群众路线教育实践活动成果，推动作风建设常态化长效化，进一步强化忧患意识、责任意识、宗旨意识，全面提高创新力、公信力、执行力，努力建设为民务实清廉、人民满意的政府。

坚持为民执政。始终把人民利益摆在最高位置，时刻把群众安危冷暖放在心中，认真化解民困、民忧，努力实现民乐、民安。

加快转变政府职能，创新服务理念，改进服务方式，优化工作流程，推行一口受理、限时办理、规范办理、透明办理和网上办理，为群众提供更加便捷的优质服务。坚持面对面解决问题、实打实为民服务，把更多人力用到公共服务上，把更多精力放到社会治理上，把更多财力投入到民生改善上。持续深化企业服务活动，集中整治企业周边环境、重点项目建设环境，深入开展“双整双促双承诺”、涉企涉民科室评议、“百姓问政”等活动，认真做好市长热线和政风行风热线办理工作，唱响优化环境“好声音”，凝聚干事创业“正能量”。

严格依法行政。忠实履行宪法和法律赋予的职责，把政府工作全面纳入法治轨道。坚决执行人大及其常委会的决议决定，自觉接受人大法律监督、工作监督和政协民主监督，认真办理人大代表议案、建议和政协提案，广泛听取民主党派、工商联、无党派人士和人民团体意见。加强行政监察和审计监督，虚心接受社会监督和舆论监督。健全依法科学民主决策机制，加强决策合法性审查，推行政府法律顾问制度，落实重大决策终身责任追究制度。提高行政执法水平，完善行政复议制度，深化法治宣传教育，做好法律援助工作。深入推进政务公开，及时回应社会关切，切实提高信息透明度、公众参与度、人民满意度。

务实高效施政。坚持求真务实，说实话、办实事，不摆花架子，不做表面文章，不搞劳民伤财的形象工程、政绩工程。高树标杆，勇争一流，大胆创新，力求每一项工作、每一件事情都做精、做细、做圆满。敢于担当，勇于负责，遇到矛盾和问题，不回避、不推诿、不退缩，在重点工作、难点事务、关键问题上，大员上阵，靠前指挥，亲力亲为。

深入基层调查研究，深入一线了解实情，倾听百姓声音，掌握一手资料。进一步精简会议、文件，大力推进电子政务。强化督查落实，狠抓跟踪问效，部署的工作快速推进，认准的事情一抓到底，承诺的事项坚决兑现，切实做到雷厉风行、快捷高效、政令畅通。

清正廉洁从政。认真落实廉政建设责任制和主体责任，强化“一岗双责”，严格规范权力运行，切实加强廉政教育，牢牢守住廉洁底线，坚决纠正损害群众利益的不正之风，严厉查处各类违纪违法行为。大力弘扬艰苦奋斗的作风，厉行勤俭节约，反对铺张浪费，力戒奢靡之风，严格控制“三公”经费。切实加强公务员队伍建设，坚决纠正不作为、乱作为，坚决克服懒政、怠政，坚决惩处失职、渎职，树立勤政廉政、干净干事的政府形象。

各位代表!困难和挑战考验着我们，责任和使命激励着我们。让我们在市委的坚强领导下，奋力拼搏、奋发作为、奋勇争先，共同谱写漯河发展新篇章!
